# Pan-enteric Capsule Endoscopy in Crohn’s Disease: Impact on Therapeutic Decisions and Inter-observer Agreement in a Multicentre Case-based Study

**DOI:** 10.1055/a-2877-2224

**Published:** 2026-06-03

**Authors:** Carlo Calabrese, Nikolas Dussias, Fernando Rizzello, Paolo Gionchetti, Alessandro Armuzzi, Marco Daperno, Antonio Rispo, Chiara Ricci, Francesco Simone Conforti, Patrizia Alvisi, Erasmo Miele, Matteo Bramuzzo, Rossella Propato, Salvatore Oliva, Gian Eugenio Tontini

**Affiliations:** 1 Scientific DirectorateIRCCS Centro di Riferimento Oncologico della BasilicataRionero In VulturePotenzaItaly; 2 Department of Medical and Surgical Sciences, IBD Unit 18508 IRCCS Azienda Ospedaliero-Universitaria di Bologna Policlinico di Sant’OrsolaBolognaEmilia-RomagnaItaly; 3 IBD Unit, Department of Gastroenterology 9268 IRCCS Humanitas Research HospitalRozzanoLombardiaItaly; 4 Department of Biomedical SciencesHumanitas UniversityMilanoItaly; 5 Gastroenterology UnitAO Ordine MaurizianoTorinoItaly; 6 Gastroenterology, Department of Clinical Medicine and SurgerySchool of Medicine “Federico II” of NaplesNaplesCampaniaItaly; 7 Gastroenterology Unit, Department of Clinical and Experimental Sciences 9297 University of BresciaBresciaLombardyItaly; 8 Gastroenterology and Endoscopy Unit 9339 Fondazione IRCCS Ca’ Granda Ospedale Maggiore PoliclinicoMilanLombardyItaly; 9 Department of Medical and Surgical SciencesIBD Unit- IRCCS Azienda Ospedaliero Universitaria- Policlinico Sant'Orsola-MalpighiBolognaEmilia-RomagnaItaly; 10 Department of Translational Medical Science, Section of Paediatrics 9307 University of Naples Federico IINaplesCampaniaItaly; 11 Institute for Maternal and Child Health 18705 IRCCS Materno Infantile Burlo GarofoloTriesteFriuli-Venezia GiuliaItaly; 12 Pediatric Gastroenterology and Liver Unit 9311 University of Rome La SapienzaRomeLazioItaly

**Keywords:** endoscopy small bowel, inflammatory bowel disease, endoscopy lower GI tract, other focus (of reviewers), GI pathology

## Abstract

**Background and study aims** Pan-enteric capsule endoscopy (CE) provides a comprehensive mucosal assessment of both the small bowel and colon in Crohn’s disease (CD). However, its incremental impact on structured clinical decision-making and inter-observer agreement remains insufficiently defined. We aimed to evaluate whether the availability of CE findings influences therapeutic decisions, risk stratification and monitoring strategies in patients with CD.

**Patients and methods** We performed a multicentre, retrospective, paired case-based study including 50 real-world CD cases (35 adults, 15 paediatric). For each case, two anonymised vignettes were generated: one incorporating clinical, biochemical and cross-sectional imaging data without CE, and one additionally including CE findings. Ten experienced inflammatory bowel disease (IBD) gastroenterologists (six adult, four paediatric) independently reviewed all vignettes in randomised order using a structured questionnaire. The primary outcome was change in therapeutic decision-making after disclosure of CE findings. Secondary outcomes included changes in risk stratification, assessment of treatment efficacy, timing of follow-up, confidence in decision-making and inter-observer agreement.

**Results** Access to CE findings significantly modified risk assessment and treatment selection. Overall, 53.3% of risk-stratification responses and 56.7% of treatment decisions changed, with a consistent shift towards higher perceived risk and treatment escalation ( *p* < 0.0001 for both). CE also altered monitoring strategies, increasing reliance on endoscopic/CE-based assessment (change rate 36.7%; *p* < 0.0001), and modestly shortened planned follow-up intervals (change rate 18.5%; *p* = 0.0366). Confidence scores showed no significant overall shift ( *p* = 0.2700), despite 41.6% of individual ratings changing. Inter-observer agreement improved from fair to moderate across several domains when CE results were available. No cases with isolated colonic CD were included in the final case set, and no capsule retention occurred in the included cohort.

**Conclusions** In this multicentre paired case-based study, pan-enteric CE substantially influenced risk stratification, treatment selection and monitoring plans in CD, while improving inter-observer agreement across several decision domains. These findings indicate that CE meaningfully affects structured clinical decision-making in selected patients, particularly when small-bowel involvement is suspected or when conventional investigations are discordant. Prospective longitudinal studies are needed to determine whether CE-guided decisions translate into improved clinical outcomes.

## Summary Box

### What is already known about this subject?

- Capsule endoscopy (CE) has higher sensitivity than cross-sectional imaging and ileocolonoscopy for detecting small-bowel Crohn’s disease and can upstage disease extent, particularly in proximal segments.
- Observational series suggest that CE findings may lead to changes in management in a relevant proportion of patients with established Crohn’s disease.
- The specific impact of CE on structured clinical decisions across multiple expert readers, and on inter-observer agreement, has not been systematically evaluated.

### What are the new findings of this study?

- In a paired pre–post, multicentre case-based design including 50 real-world Crohn’s disease cases (35 adults, 15 paediatric), adding CE led to changes in 53.3% of risk-stratification responses and 56.7% of treatment decisions made by 10 experienced IBD gastroenterologists.
- Availability of CE findings systematically shifted management towards higher perceived disease risk, more frequent treatment escalation (particularly biologic initiation or intensification) and greater reliance on endoscopic/CE-based monitoring.
- Inter-observer agreement improved from fair to moderate across several decision domains when CE was available, suggesting that CE not only changes decisions but also makes them more consistent between clinicians.
- CE had a modest effect on the timing of follow-up and did not produce a net increase in self-reported confidence at group level, despite prompting re-evaluation of confidence in approximately 40% of assessments.

## Introduction

Crohn’s disease (CD) is a chronic, relapsing-remitting inflammatory condition that can involve any segment of the gastrointestinal tract and is associated with cumulative bowel damage, hospitalisation and a substantial need for surgical intervention within the first decade after diagnosis. 1 Modern management increasingly adopts a treat-to-target strategy, requiring accurate assessment of disease extent and activity to guide early risk stratification and timely optimisation of therapy. 2

Diagnosis and monitoring of CD rely on an integrated approach combining clinical assessment, biomarkers, cross-sectional imaging and endoscopy. 2 3 4 Ileocolonoscopy remains the reference standard for direct visualisation and histological sampling of the colon and terminal ileum, but it is invasive, resource-intensive and cannot assess proximal small-bowel segments. 3 Cross-sectional techniques such as MR enterography and CT enterography provide transmural and extra-intestinal information, yet they may miss subtle mucosal lesions and are less frequently used for repeated monitoring in routine practice. 4 5 6

Capsule endoscopy (CE) is a minimally invasive tool that offers high-resolution visualisation of the entire small bowel and, with pan-enteric devices, the colon as well. 7 Multiple studies and meta-analyses have shown that CE has higher sensitivity than cross-sectional imaging for early or proximal small-bowel CD, and can upstage disease extent compared with ileocolonoscopy and radiology, particularly in patients with suspected or established small-bowel involvement. 7 8 9 10 Current European guidelines recognise CE as a valuable modality in selected CD scenarios, while recommending careful pre-procedure assessment for strictures and the use of a patency capsule before small-bowel CE in patients with established CD to reduce capsule retention risk. 11 12 13

Despite these advantages, the clinical impact of CE in CD management remains incompletely defined. Previous studies have largely focused on diagnostic yield, disease upstaging or crude management changes after CE in single-centre cohorts. However, less is known about the incremental effect of CE on structured therapeutic and monitoring decisions when assessed in a controlled paired design, and about whether the availability of CE findings reduces variability between expert clinicians. We therefore conducted a multicentre paired case-based study using real-world CD scenarios presented with and without CE findings to evaluate the impact of pan-enteric CE on risk stratification, therapeutic decisions, monitoring strategies and inter-observer agreement among experienced adult and paediatric IBD gastroenterologists.

## Materials and Methods

### Study Design and Case Selection

We conducted a retrospective, multicentre, case-based study including 50 real-world CD cases drawn from tertiary adult and paediatric IBD centres. Eligible cases were consecutive patients with an established diagnosis of CD who had undergone pan-enteric CE as part of routine clinical care. Pan-enteric rather than small-bowel CE was selected because the purpose of the study was to assess the incremental impact of whole-gut mucosal information on clinical decision-making, including both small-bowel and colonic disease assessment. For each patient, a de-identified clinical vignette was constructed summarising demographics, relevant medical history, prior and current therapies, clinical presentation, biochemical markers and conventional investigations available in the same clinical work-up, including cross-sectional imaging and ileocolonoscopy when available. Ileocolonoscopy and cross-sectional imaging (MRE and/or CTE) were available within four weeks of CE and were summarised in the baseline vignette.

### Readers and Evaluation Procedure

Ten experienced gastroenterologists with recognised expertise in IBD (adult and paediatric) participated as independent readers. Each reader assessed all cases. Vignettes were presented in random order with a washout period of at least two intervening cases between the baseline and CE-augmented version of the same patient to minimise recall bias. Readers were blinded to the identity of the patients, to each other’s responses and to the sequence in which case versions were presented.

For each case, two versions of the vignette were generated: (1) a baseline vignette including clinical, laboratory and conventional imaging data without CE, and (2) a CE-augmented vignette including the same information plus a standardised description of CE findings, including lesion location, extent, severity, and the presence of complications. The aim of the study was to evaluate the impact of CE information on downstream clinical decision-making rather than to assess primary video interpretation accuracy by the readers. Several readers also had prior experience in CE interpretation.

After each vignette, readers completed a structured questionnaire addressing five key decision domains:

1. Risk stratification – overall disease risk (e.g. low, intermediate or high), considering current activity and long-term prognostic factors.
2. Treatment selection – initial therapeutic strategy (e.g. no change, optimisation of current therapy, introduction or escalation of immunomodulators or biologics, combination therapy, or de-escalation).
3. Assessment of treatment efficacy – preferred modality to evaluate response (clinical indices, biomarkers, cross-sectional imaging, CE or colonoscopy).
4. Timing of follow-up – planned interval to reassess the patient (categorical options covering short, intermediate and longer-term follow-up).
5. Confidence in decision-making – self-reported confidence in the chosen management plan, rated on a numerical scale.

The questionnaire was identical for the baseline and CE-augmented versions of each case.

### Statistical Analysis

Descriptive statistics were used to summarise the distribution of responses for each question with and without CE. The primary outcome was the proportion of decisions that changed between the baseline and CE-augmented vignette for each question (Q1–Q5).

Paired categorical responses were compared using the Stuart–Maxwell test of marginal homogeneity.

Inter-observer agreement across readers was quantified using Fleiss’ kappa with 95% confidence intervals for each decision domain, separately for the condition without CE and with CE. Kappa values were interpreted using conventional thresholds (e.g. <0.20 poor, 0.21–0.40 fair, 0.41–0.60 moderate).

For exploratory purposes, we also fitted logistic mixed-effects models to estimate the odds of any decision change after adding CE, with random effects for reader and case to account for clustering. Odds ratios (ORs) and 95% confidence intervals were summarised overall and by decision domain. All analyses were conducted using R and RStudio.

The study was performed in accordance with the Declaration of Helsinki. The protocol was approved by the local ethics committee, which granted a waiver of written informed consent given the fully anonymised, retrospective nature of the data.

## Results

### Study Population and Capsule Endoscopy Findings

Fifty anonymised CD cases (35 adults and 15 paediatric patients) were included in the analysis. CE characteristics of the case set are summarised in Table 2 . According to the Montreal classification, most cases were A2 at diagnosis (76%), with A1 and A3 accounting for 10% and 14%, respectively.

Behavioural phenotypes were evenly distributed between B1 (38%) and B2 (40%), while 22% of patients had penetrating disease (B3).

In terms of disease location, 44% of cases were classified as L1 (small bowel) and 56% as L3 (ileocolonic). On CE, lesions were predominantly located in the small bowel (L1) in 38% of cases and in ileocolonic segments (L3) in 62%. Ulcerative lesions were present in 86% of cases, while scars without active ulceration were observed in 14%. The median Lewis score was 585 (interquartile range [IQR] 337-1104), and any degree of stenosis on CE was recorded in 32% of patients. Overall, this profile is consistent with a cohort enriched for small-bowel and ileocolonic CD with a high burden of ulcerative mucosal disease. No cases with isolated colonic CD (Montreal L2) were included in the final case set; patients were classified as L1 or L3. No cases of capsule retention occurred in the included cohort.

### Overall Decision Changes and Inter-observer Agreement

**Fig. 1:**
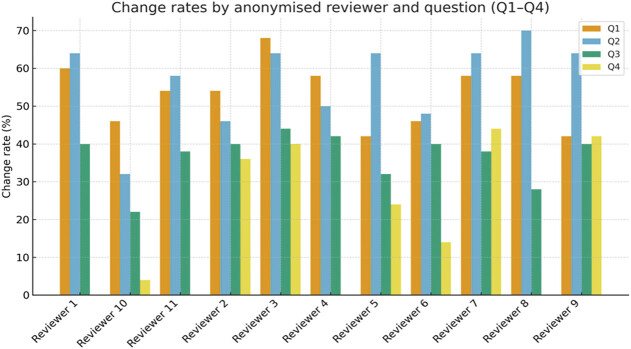
Per-reader change rates for Q1–Q4 in the paired pre–post experiment. Each point represents one IBD expert (adult or paediatric). For each decision domain (Q1–Q4), the *y* -axis shows the percentage of responses that changed after capsule endoscopy (CE) findings were added. Vertical lines indicate median and interquartile range across the 10 readers.

**Fig. 2:**
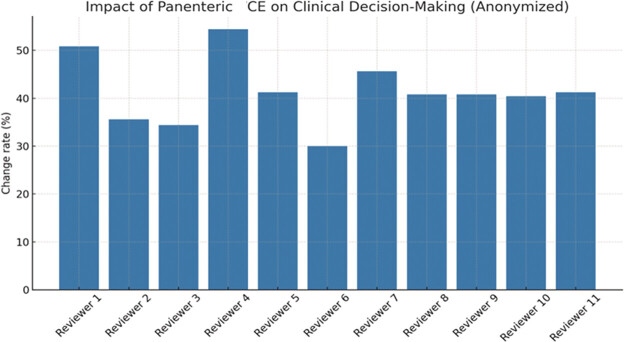
Overall percentage of clinical decisions modified by capsule endoscopy for each reader. Each bar represents the proportion of all decisions (Q1–Q5) that changed when CE findings were incorporated compared with the same cases assessed without CE. The dashed horizontal line indicates the median change rate across readers.

A total of 500 paired evaluations were available for each decision domain (Q1–Q5) across the 10 expert readers and 50 cases. Table 1 summarises the percentage distribution of responses with and without CE, while per-reader change rates for each question are shown in Fig. 1 . The overall proportion of decisions modified by CE for each reader is depicted in Fig. 2 .

**Table 1:** Distribution of responses with and without capsule endoscopy.

Question	Response option	Without CE (%)	With CE (%)
How would you stratify this patient’s risk for aggressive/complicated disease?	Very low risk	2.9%	1.6%
	Low risk	25.1%	14.9%
	Moderate risk	34.5%	34.5%
	High risk	29.5%	37.5%
	Very high risk	8.0%	11.5%
What primary treatment would you recommend for this patient?	Mesalamine / no treatment	17.3%	8.9%
	Systemic steroids	11.3%	8.5%
	Immunosuppressants	4.4%	4.2%
	Start biologic therapy	28.5%	41.6%
	Continuing current biologic	12.0%	6.2%
	Intensify current biologic	7.3%	9.8%
	De-escalate current biologic	1.1%	0.4%
	Switch biologic class	12.2%	16.7%
	Other	6.0%	3.6%
How would you assess treatment efficacy?	Clinical and laboratory response	19.1%	9.3%
	Cross-sectional imaging/ultrasound	21.8%	13.5%
	Endoscopy/capsule endoscopy	59.1%	77.3%
When would you evaluate treatment efficacy?	Only if needed	7.5%	4.9%
	At 6 months	54.9%	56.2%
	At 12 months	37.6%	38.9%
What is your confidence level in the previous answers?	Very low confidence	0.2%	0.2%
	Low confidence	6.0%	4.2%
	Moderate confidence	45.8%	45.8%
	High confidence	43.3%	46.4%
	Very high confidence	4.7%	3.5%

**Table 2:** Capsule endoscopy characteristics of the 50 Crohn’s disease cases used in the case-based experiment.

Parameter	Value
**Number of cases**	**50**
**Age at diagnosis, Montreal**	
A1 (<=16 years)	5 (10.0%)
A2 (17-40 years)	38 (76.0%)
A3 (>40 years)	7 (14.0%)
**Behaviour, Montreal**	
B1 (non-stricturing, non-penetrating)	19 (38.0%)
B2 (stricturing)	20 (40.0%)
B3 (penetrating)	11 (22.0%)
**Location, Montreal**	
L1 (small bowel)	22 (44.0%)
L3 (ileocolonic)	28 (56.0%)
**Capsule endoscopy lesion site**	
Small bowel (L1)	19 (38.0%)
Ileocolonic (L3)	31 (62.0%)
**Lesion type on CE**	
Ulcerative lesions	43 (86.0%)
Scars	7 (14.0%)
**Lewis score**	**Median 585 (IQR 337–1104)**
**Stenosis on CE (any)**	**16 (32.0%)**

**Fig. 3:**
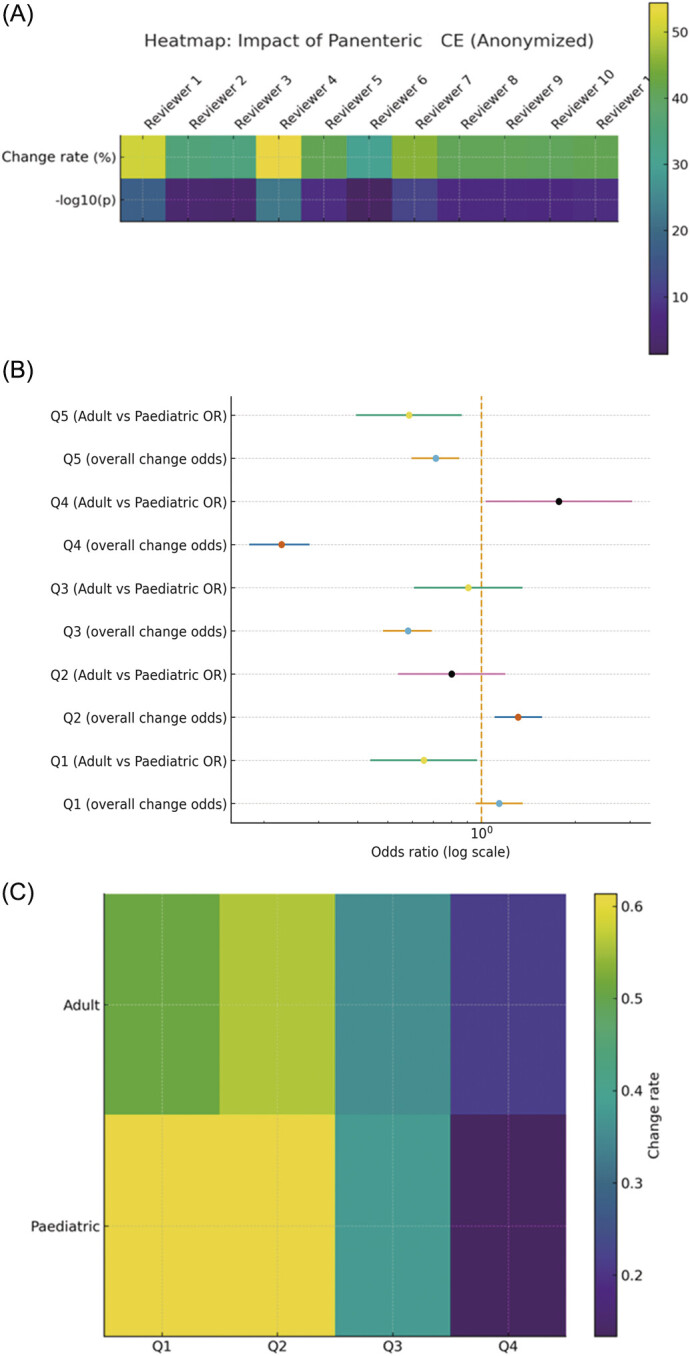
Impact of pan-enteric capsule endoscopy in the multi-reader experiment. ( **A** ) Heatmap summarising the impact of CE across reviewers and decision domains (Q1–Q4). Columns represent anonymised reviewers and rows represent the questions. Cell colour encodes both the percentage of decisions that changed and the statistical significance of the paired comparison (−log10 *p* value). ( **B** ) Forest plot showing effect sizes (odds of any decision change) across Q1–Q4, derived from logistic mixed-effects models, with 95% confidence intervals. Where estimable, subgroup odds ratios for adult and paediatric readers are displayed. ( **C** ) Change-rate heatmap stratified by reader group (adult vs paediatric) and decision domain (Q1–Q4); warmer colours indicate higher proportions of decisions modified by CE.

In the paired pre–post framework, decision changes were frequent across all domains. A global overview of the impact of CE across decision domains and reviewers is provided by the heatmap in Fig. 3A . In exploratory logistic mixed-effects models, the odds of any decision change were consistently elevated across questions Q1–Q4, with effect sizes illustrated in the forest plot in Fig. 3B . Change-rate patterns stratified by adult and paediatric readers are displayed in Fig. 3C .

### Question 1 – Risk Stratification

For overall risk stratification (Q1), the addition of CE information significantly altered the distribution of risk categories (Stuart–Maxwell statistic = 44.819; df = 4; *p* < 0.0001).

Overall, 53.3% of individual risk-stratification responses differed between the baseline and CE-augmented vignettes. Readers tended to reclassify patients towards higher risk categories when CE revealed more extensive or severe small-bowel involvement than suggested by conventional investigations. Inter-observer agreement for risk stratification improved from fair (Fleiss’ kappa = 0.21) without CE to moderate (kappa = 0.42) with CE.

### Question 2 – Treatment Selection

For treatment selection (Q2), CE had an even more pronounced impact. The distribution of chosen therapeutic strategies changed significantly after CE (Stuart–Maxwell statistic = 92.525; df = 8; *p* < 0.0001), with 56.7% of responses being altered. Decisions shifted predominantly towards treatment escalation, including initiation or intensification of biologic therapy, particularly in cases where CE demonstrated more extensive or severe mucosal inflammation. Inter-observer agreement for treatment selection improved from kappa = 0.32 (fair) without CE to kappa = 0.47 (moderate) when CE findings were available.

### Question 3 – Assessment of Treatment Efficacy

Regarding the preferred modality to assess treatment response (Q3), CE also significantly influenced decision-making (Stuart–Maxwell statistic = 106.011; df = 2; *p* < 0.0001). The proportion of responses that changed was 36.7%. In the CE-augmented condition, readers more frequently selected endoscopic or capsule-based assessment as their primary tool for monitoring efficacy, rather than relying solely on clinical indices, biomarkers or cross-sectional imaging. Inter-observer agreement improved from kappa = 0.46 without CE to kappa = 0.61 with CE, indicating a shift from moderate towards substantial concordance.

### Question 4 – Timing of Follow-up

The timing of planned follow-up (Q4) was less affected by CE. Although the distribution of follow-up intervals changed modestly (Stuart–Maxwell statistic = 6.615; df = 2; *p* = 0.0366), the overall proportion of altered responses was relatively low (18.5%). When changes occurred, they generally reflected a shorter intended interval in the presence of extensive or severe CE findings. Fleiss’ kappa for follow-up timing remained in a similar range, increasing only slightly from 0.38 without CE to 0.41 with CE.

### Question 5 – Confidence in Decision-making

Self-reported confidence in the management plan (Q5) showed no statistically significant shift at group level (Stuart–Maxwell statistic = 5.173; df = 4; *p* = 0.2700). Nevertheless, 41.6% of confidence ratings differed between the baseline and CE-augmented vignettes, indicating that CE information frequently prompted clinicians to reconsider how secure they felt about their decisions. Inter-observer agreement for confidence ratings remained in the fair range for both conditions (kappa = 0.25 without CE vs kappa = 0.27 with CE).

## Discussion

This multicentre paired case-based study shows that pan-enteric CE meaningfully influenced structured therapeutic decision-making in CD. When CE findings were added to conventional clinical, biochemical and cross-sectional imaging data, more than half of risk-stratification and treatment responses were modified by experienced IBD specialists. Decisions shifted consistently towards higher perceived disease risk, greater use of biologics or treatment escalation and closer endoscopic monitoring. At the same time, inter-observer agreement improved from fair to moderate across several domains when CE information was available. These results support the clinical utility of CE as a complementary tool for whole-gut mucosal assessment beyond conventional imaging techniques. 1 2 7 8 9 10

Our observations are in line with the evolving role of CE in CD. Ileocolonoscopy and cross-sectional imaging remain the cornerstones of phenotyping and monitoring, 2 3 4 11 but they may miss subtle or proximal small-bowel lesions, especially in L4 disease or when inflammation is patchy. 3 4 5 6 8 9 Several studies and meta-analyses have shown that small-bowel CE has higher sensitivity than MR enterography, CT enterography or small-bowel ultrasound for detecting early inflammatory changes, and can upstage disease extent compared with ileocolonoscopy and radiology. 1 7 8 9 10 Current ECCO and ESGE guidelines therefore position CE as a key modality in selected CD scenarios, provided that the risk of strictures and capsule retention is carefully assessed and, where appropriate, patency testing is used. 11 12 13 14 15 16 Standardised CE activity indices and CE-based studies, including the Lewis score and the Capsule Endoscopy Crohn’s Disease Activity Index, support quantification of small-bowel mucosal inflammation and underscore the clinical relevance of capsule-detected lesions in established CD. 17 18 19 20 In our cohort, systematically adding CE data appears to function as a “second look” at the mucosa, prompting upward reclassification of risk and more proactive therapeutic strategies in a substantial proportion of cases. This aligns with treat-to-target recommendations, which emphasise the importance of detecting ongoing subclinical activity to prevent long-term complications. 21 22

The impact of CE was most evident on decisions related to disease aggressiveness and treatment selection, whereas the effect on timing of follow-up and assessment of treatment response was more modest. Several explanations are plausible. First, follow-up intervals and judgements about treatment efficacy are influenced by a broader set of factors, including prior disease course, comorbidities and patient preferences, which are not fully captured in vignette-based scenarios. Second, clinicians may integrate CE findings with longitudinal trends in symptoms and biomarkers rather than using a single imaging result to redefine monitoring intervals. Nevertheless, the observed shift towards endoscopy- or CE-based monitoring is consistent with data showing that mucosal disease activity assessed by CE correlates with future outcomes and may be an appropriate target for therapy in small-bowel-predominant CD. 18 19 20 23 24 25

Interestingly, the substantial changes in management were not accompanied by a marked increase in self-reported confidence. Confidence scores changed in around 40% of evaluations but did not show a significant net increase overall. This apparent dissociation between altered decisions and relatively stable confidence may reflect how experienced clinicians integrate new information. Gastroenterologists accustomed to managing complex CD may adjust therapeutic plans when confronted with additional mucosal data while remaining cautious in their global judgement, especially in the absence of histology or long-term outcome data. Similar patterns have been described in other multireader studies where advanced imaging improves diagnostic yield and standardises reporting without necessarily modifying physicians’ subjective certainty. 8 9 23

From a methodological standpoint, the paired pre–post design is a major strength of this study. Presenting each real-world case twice, with and without CE data, allowed us to isolate the specific contribution of CE while controlling for inter-individual variability among readers. The inclusion of multiple experts from both adult and paediatric IBD units enhances the generalisability of the findings across different practice settings. 8 10

Importantly, our data suggest that CE reduces variability between clinicians, with kappa values moving from fair to moderate when CE findings were available. Inter-observer variability is a recognised challenge in endoscopic assessment and disease-activity scoring, 12 13 24 and any tool that helps to standardise interpretation is highly relevant for routine practice.

This study has several limitations. First, its retrospective paired case-based design was intended to evaluate the incremental impact of CE information on structured decision-making, but it does not allow conclusions on whether CE-guided management improves long-term clinical outcomes such as corticosteroid-free remission, hospitalisation, surgery or treatment persistence. Second, although vignettes were built from real-world cases and included conventional investigations available in routine care, the diagnostic work-up was not fully standardised across all patients within a uniform predefined time window. Third, the final case set was enriched for small-bowel and ileocolonic disease and did not include isolated colonic CD, which limits generalisability to L2 phenotypes. Fourth, the mixed adult and paediatric reader panel was intentionally chosen to explore the consistency of CE-driven decision changes across experienced IBD clinicians, but cross-specialty assessment may have introduced additional variability related to age-specific practice patterns. Fifth, readers were provided with standardised CE findings rather than being asked to independently interpret raw capsule videos; therefore, the study does not address the effect of different levels of capsule-reading expertise on downstream management choices. Finally, although CE may support closer mucosal monitoring in selected patients, repeated use in CD must remain individualised because retention risk is not negligible, particularly in established or stricturing disease. 18 19 20 21 25 26 27

Despite these limitations, our findings add important evidence to the expanding literature on CE in IBD. Previous series have primarily focused on the diagnostic yield of CE or on the proportion of patients in whom management was changed after CE in single-centre cohorts. 19 20 21 26 27 The present study complements those data by examining structured multi-reader decisions and inter-observer agreement in a paired design. In practical terms, our results suggest that CE may be particularly useful in scenarios where conventional imaging is inconclusive or discordant with symptoms, in patients with suspected small-bowel involvement beyond the reach of ileocolonoscopy and in complex postoperative or multi-operated cases. 7 8 9 10 18 19 20 23 In such settings, identifying additional areas of active mucosal disease can reasonably justify therapeutic escalation and a more intensive, endoscopy-based monitoring strategy.

Future research should now move towards prospective, longitudinal designs in which CE-guided decisions are linked to hard outcomes such as corticosteroid-free remission, mucosal healing, hospitalisation, surgery and treatment persistence. 18 19 20 21 25 26 27 It will also be important to explore how CE can be integrated into multimodal treat-to-target algorithms alongside cross-sectional imaging, biomarkers and clinical scores, and whether structured reporting or scoring systems can further reduce inter-observer variability. 12 13 24 25 Finally, emerging approaches such as automated lesion detection and artificial intelligence-based activity scoring may, in the future, complement expert interpretation and further standardise CE-driven decision-making, but these strategies require rigorous validation in dedicated studies. 11

In conclusion, this multicentre paired case-based study shows that pan-enteric CE substantially influences risk assessment, therapeutic strategies and monitoring plans in CD, while improving agreement between experienced IBD clinicians across several decision domains. Rather than demonstrating improved patient outcomes, our findings show that CE provides clinically relevant additional information that materially changes structured management decisions beyond standard clinical, biochemical and cross-sectional data. These results support the selective integration of CE into the evaluation of patients with suspected small-bowel involvement or discordant conventional investigations, while prospective longitudinal studies are needed to determine whether CE-guided decisions translate into better long-term outcomes.

## Data Availability

De-identified individual-level data underlying the results presented in this article, together with the study protocol and statistical analysis plan, are available from the corresponding author upon reasonable request for research purposes, subject to institutional approvals and GDPR-compliant data-sharing agreements. No public repository is used owing to patient-privacy and institutional policy constraints.
